# *Mycoplasma pneumonia* With Cavitary Lesions

**DOI:** 10.1002/ccr3.71186

**Published:** 2025-10-06

**Authors:** Ikkei Oyama, Kosuke Ishizuka, Takuya Otsuki, Iori Motohashi, Kenya Ie, Chiaki Okuse

**Affiliations:** ^1^ Department of General Internal Medicine Kawasaki Municipal Tama Hospital Kawasaki Kanagawa Japan; ^2^ Department of General Internal Medicine St. Marianna University School of Medicine Kawasaki Kanagawa Japan; ^3^ Department of General Medicine Yokohama City University School of Medicine Yokohama Kanagawa Japan; ^4^ Department of General Medicine Yokohama City University Medical Center Yokohama Kanagawa Japan

**Keywords:** case report, cavitary lung lesions, community‐acquired pneumonia, computed tomography, differential diagnosis, *Mycoplasma pneumonia*

## Abstract

*Mycoplasma pneumoniae* usually causes mild disease but can rarely present with cavitary lesions. Recognizing this atypical finding is important to avoid misdiagnosis of serious infections.

## 1 Case

**FIGURE 1:**
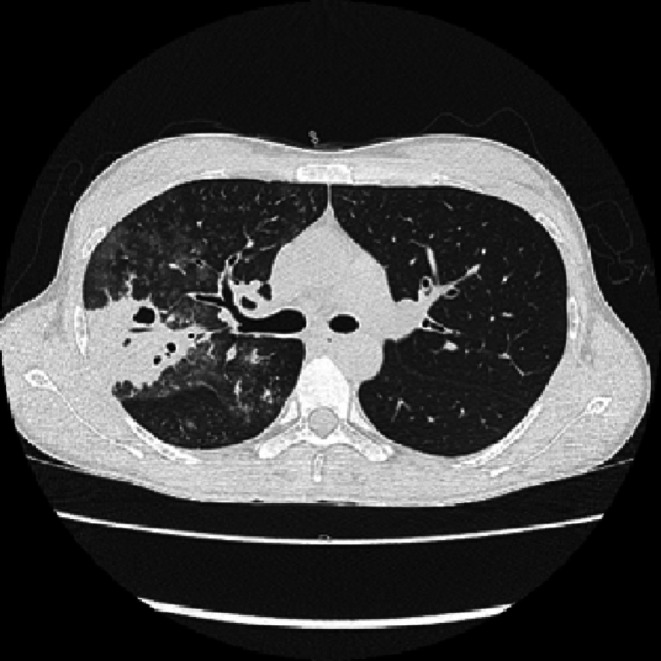
CT revealed consolidation, ground‐glass opacities, and cavitary lesions predominantly in the right upper lobe.

**FIGURE 2:**
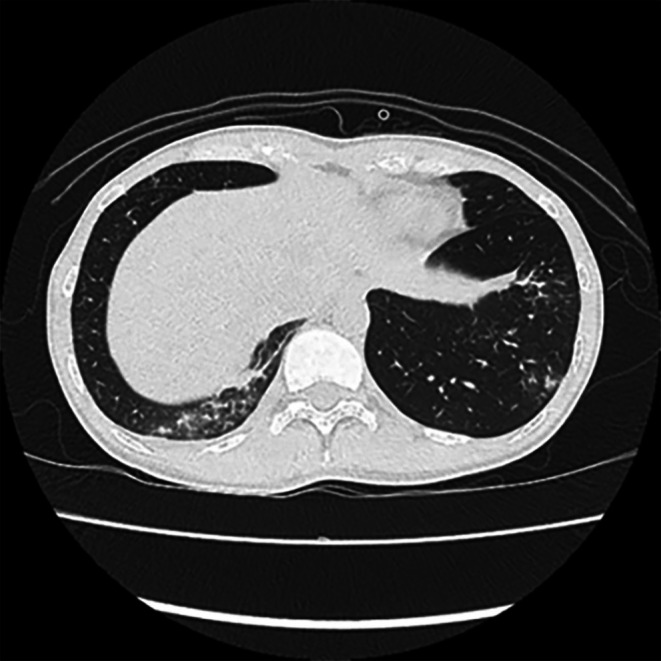
CT revealed consolidation and ground‐glass opacities in both lower lobes.

A 33‐year‐old Japanese woman presented with a 10‐day history of fever and cough. She had no significant past medical history and was not taking any medications. Her vital signs were a temperature of 37.5°C, a pulse rate of 93 beats/min, blood pressure of 93/65 mmHg, and oxygen saturation of 96% on room air. Physical examination revealed coarse crackles in both lower lung fields, predominantly on the right side. Laboratory tests showed a white blood cell (WBC) count of 5700/μL, aspartate aminotransferase (AST) of 53 U/L, alanine aminotransferase (ALT) of 42 U/L, lactate dehydrogenase (LDH) of 368 U/L, and C‐reactive protein (CRP) of 9.06 mg/dL. A rapid *Mycoplasma* antigen test was positive. Chest computed tomography (CT) revealed consolidation, ground‐glass opacities, and cavitary lesions predominantly in the right upper lobe (Figures 1 and 2). Based on these findings, the patient was diagnosed with pneumonia caused by *Mycoplasma pneumoniae* . Minocycline therapy was initiated, resulting in rapid defervescence, and the patient was discharged on hospital day 11. The sputum culture test for general bacteria and acid‐fast bacteria for three consecutive days, as well as the interferon gamma release assay for tuberculosis, were negative. One month later, during outpatient follow‐up, there was no recurrence of symptoms, and chest CT confirmed the resolution of cavitary lesions.

*Mycoplasma* infections are caused by droplet transmission of bacteria belonging to the genus *Mycoplasma* and are globally recognized as a leading cause of primary atypical pneumonia [1]. Common symptoms include sore throat, fever, and cough, while extrapulmonary complications such as encephalitis and myelitis may occur in some cases [1]. In Mycoplasma pneumonia, chest CT typically reveals findings such as consolidation, ground‐glass opacities, nodular shadows, and bronchovascular thickening [2]. However, as in this case, cavitary lesions caused by Mycoplasma pneumonia have also been reported [3].

## Author Contributions

**Ikkei Oyama:** conceptualization, data curation, investigation, writing – original draft. **Kosuke Ishizuka:** writing – review and editing. **Takuya Otsuki:** writing – review and editing. **Iori Motohashi:** writing – review and editing. **Kenya Ie:** writing – review and editing. **Chiaki Okuse:** writing – review and editing.

## Consent

Written informed consent was obtained from the patient for the publication of this case report and accompanying images.

## Conflicts of Interest

The authors declare no conflicts of interest.

## Data Availability

Data sharing not applicable to this article as no datasets were generated or analyzed during the current study.
